# Utilizing a responsive web portal for studying disc tracing agreement in retinal images

**DOI:** 10.1371/journal.pone.0251703

**Published:** 2021-05-25

**Authors:** Abdullah Sarhan, Andrew Swift, Adam Gorner, Jon Rokne, Reda Alhajj, Gavin Docherty, Andrew Crichton

**Affiliations:** 1 Department of Computer Science, University of Calgary, Calgary, Canada; 2 Cumming School of Medicine, University of Calgary, Calgary, Canada; 3 Department of Computer Engineering, Istanbul Medipol University, Istanbul, Turkey; 4 Department of Health Informatics, University of Southern Denmark, Odense, Denmark; 5 Department of Ophthalmology and Visual Sciences, University of Calgary, Calgary, Canada; University of Oklahoma, UNITED STATES

## Abstract

Glaucoma is a leading cause of blindness worldwide whose detection is based on multiple factors, including measuring the cup to disc ratio, retinal nerve fiber layer and visual field defects. Advances in image processing and machine learning have allowed the development of automated approached for segmenting objects from fundus images. However, to build a robust system, a reliable ground truth dataset is required for proper training and validation of the model. In this study, we investigate the level of agreement in properly detecting the retinal disc in fundus images using an online portal built for such purposes. Two Doctors of Optometry independently traced the discs for 159 fundus images obtained from publicly available datasets using a purpose-built online portal. Additionally, we studied the effectiveness of ellipse fitting in handling misalignments in tracing. We measured tracing precision, interobserver variability, and average boundary distance between the results provided by ophthalmologists, and optometrist tracing. We also studied whether ellipse fitting has a positive or negative impact on properly detecting disc boundaries. The overall agreement between the optometrists in terms of locating the disc region in these images was 0.87. However, we found that there was a fair agreement on the disc border with kappa = 0.21. Disagreements were mainly in fundus images obtained from glaucomatous patients. The resulting dataset was deemed to be an acceptable ground truth dataset for training a validation of models for automatic detection of objects in fundus images.

## Introduction

A study by Bourne et al. indicates that, as of 2015, there were approximately 444 million people living with visual impairments worldwide; 39 million of these were blind, 216 million had moderate to severe visual impairment, and 189 million had mild visual impairment [1]. A major contribution to these statistics comes from vision loss to glaucoma. It is the world’s second leading cause of irreversible vision loss after cataracts, accounting for 12% of cases of blindness annually [2]. It is estimated that the number of people between the ages of 40–80 affected by glaucoma will increase from the present of 80 million to 111.8 million by 2040 [3]. Furthermore, 2.4% of all individuals and 4.7% of those over 70 are at risk of developing this condition [4].

The term glaucoma refers to a condition caused by a group of diseases that leads to the degeneration of retinal ganglion cells (RGCs). The death of RGCs leads to (i) structural changes to the optic nerve head and the nerve fiber layer and (ii) simultaneous functional constriction of the visual field [4–6]. These two effects of glaucoma cause peripheral vision loss and, if left untreated, can eventually lead to blindness. One possible indicator of glaucoma is increased intraocular pressure (IOP), which can damage the RGCs, resulting in nerve fibre layer atrophy and thus structural changes to the optic nerve head.

Current approaches for glaucoma detection rely on manual interpretation of fundus images by optometrists and ophthalmologists. First patients would visit an optometrist who will then be referred to an ophthalmologist or not based on the interpretation of the fundus image. This interpretation is both critical and time-consuming and relies on the experience of the ophthalmologist/optometrist. in particular, they have difficulties in detecting the initial stages of glaucoma. As a result, about 80% of early-onset glaucoma cases may go undiagnosed [7]. Thus, there is a critical need for automated tools to accurately detect glaucoma which will then have the potential to decrease blindness due to glaucoma [7].

The advent of machine learning has opened up new possibilities for the automatic analysis of medical images and in particular for analyzing and segmenting fundus images. for the detection of various eye conditions, such as glaucoma and diabetic retinopathy [6, 8].

With the rise of deep learning comes the potential for achieving high performance when automating the segmentation of various objects from retinal images such as disc segmentation [9–11]. However, a common requirement for proper training for these models is the availability of a ground truth dataset. Creating such datasets with reliable ground truth labeling can be both subjective and time-consuming [12, 13]. Furthermore, issues of subjectivity in the labeling by experts add to the difficulty of developing the dataset. To handle the time issue researchers have been using crowdsourcing to annotate and label a large number of images, especially in telemedicine. Unfortunately, the application of this approach has not yet demonstrated that the results are reliable enough to be used as ground truths.

In the context reliable is defined in terms of several experts making the same identification of an object or objects in a fundus image. While this is a somewhat imprecise definition it reflects the underlying problem of making an absolute definition of objects in a fundus image. This problem also occurs in other deep learning approaches where it is overcome by also increasing the sizes of the datasets.

Most crowdsourcing approaches in the retinal field have focused on comparing the classification accuracy of non ophthalmologists/optometrists undergoing a brief training instead of comparing the tracing done for various anatomical retinal objects [14]. Mitry et al., [15] conducted a study utilizing the crowdsourcing technique that focuses on discriminating between normal and glaucomatous discs. Amazon’s Mechanical Turk platform was used to recruit participants known as knowledge workers (KW) from the online community [16] with the KWs that perform well being denoted as “masters”. The study obtained 2,540 classifications for 127 color fundus images within 24 hours and the performance of KWs with previous experience in performing such a task was compared to those without. The average area under curve (AUC)-a performance measure for binary classifier where a value closer to 100 indicates greater performance-achieved was 62.75%, with no significant difference between the two groups. However, specificity was very low (38.9%). Further, they did not measure the agreement between KWs. Mitry et al., [17] performed another crowdsourcing study as an extension to the previous one they conducted [15]. In this study, they found an overall sensitivity of 71% and specificity of 87% for all classifications and a masters-only group achieved the highest performance, which was 10% superior to that of the non-masters.

The study conducted by Son et al., [18], measured the accuracy of KWs recruited using Amazon Mechanical Turk in correctly localizing abnormalities in 109,985 images. However, they neither localized the disc boundaries nor the peripapillary atrophy (PPA), which are indicators of glaucoma [19]. Another study measures the performance of experienced ophthalmologists instead of KWs [20]. On average, three experienced raters agreed that an abnormality was present for 46.4% of the images, whereas two raters were in agreement for 69.9% of the images. The agreement rate when all three raters participated ranged from 5.7% to 43.3%. However, none of these approaches works when attempting to trace a disc.

None of the studies discussed above focused on comparing the tracing performed for the disc in retinal fundus images, nor did they investigate participants’ ability to discriminate between the disc border and the PPA, which is important when diagnosing glaucoma [19]. Rather, these studies mainly focused on image classification and/or identifying regions with abnormalities and did not measure the level of agreement between different groups of participants.

In this study, we investigate the tracing done by two doctors of optometry OD1 and OD2, for the retinal disc by utilizing a responsive web portal built for such purposes. This portal can also be used for the identification of other objects in the retina. Unlike other desktop applications, such as MS-Paint that work on some devices with a specific operating system, the portal can also be used on any device with access to a browser and internet, including mobile phones, tablets, laptops, and desktops. Doing so allows users to perform tracing for fundus images regardless of the type of device they are using. Additionally, we compare the tracing performed by the two optometrists with previous ones performed by ophthalmologists which are provided by the publishers of these datasets.

Exact tracing can be time-consuming, but it is required to develop a ground truth that can be used to train machine learning models for consistent and proper segmentation of retinal objects. For instance, it is important to be able to discriminate between disc boundary and PPA region when diagnosing glaucoma. Hence, the goal of this study is to investigate and compare the tracing of the disc performed by the two optometrists through the responsive online portal that we developed. To our knowledge, there is no publicly available portal similar to the one developed in this study. Our contributions can be listed as follows:

1. We demonstrated the level of agreement/disagreement between optometrists for disc tracing.
2. We demonstrated the level agreement/disagreement between ophthalmologists and optometrists.
3. We showed that using ellipse fitting for adjusting misalignments in the traced disc does not always perform well especially in the case of glaucoma.
4. We published the new tracings performed by the optometrists so that researchers can use the data as a ground truth dataset when developing their models.
5. We developed an online portal that can be used for annotating discs by multiple contributors. This portal can be expanded to other retinal object and even be used for educational purposes.

## Materials and methods

The doctors of optometry OD1 and OD2 and one ophthalmologist specializing in glaucoma were involved in this study. The two optometry doctors were responsible for tracing the disc and the glaucoma specialist investigated the images that had a high disagreement. Each optometry doctor traced the discs in retinal images independently, using their own laptops/tablets. They used the built-in features of the web portal to perform the tracing. This section discusses the datasets used, together with how the tracing was done and the statistical analysis conducted.

### Datasets

The publicly available datasets Drishti, [21], and ARIA, [22], were used in this study. A total of 159 images were used from these datasets. One of the reasons for using these datasets is that we wished to evaluate tracing variability when different retinal conditions were presented. Additionally, these datasets are the most commonly used datasets by researchers working in the field of ophthalmology and machine learning. Of the images, 69 depicted cases of glaucoma, 53 diabetic retinopathy, and 37 depicted normal cases. The normal and glaucomatous images were obtained from the Drishti dataset, while those depicting diabetic retinopathy (DR) were obtained from the ARIA dataset. The images in the ARIA dataset are taken from a 50-degree field of view and stored in “.tif” format, with dimensions of 768 × 576 pixels, while those from the Drishti dataset are taken from a 30-degree field of view and stored in “.png” format, with dimensions of 2,896 × 1,944 pixels.

Each image obtained from these datasets had its own disc ground truth provided by a specialist ophthalmologist. Hence, in our analysis, we considered the ground truth provided in these datasets as our reference if the two doctors of optometry properly trace the disc or not. When high disagreement occurred the glaucoma specialist would investigate the reason behind this disagreement. All tracings generated in this study are available online for researchers to use for further investigation [23]. Note that we did not collect any new retinal images and hence we just used those publicly available datasets and compared the tracing performed in our study to the ground truth provided in these datasets. We did not need to obtain ethics approval as this was already performed by the researchers who collected the images and made them available.

### Study design

**Fig 1:**
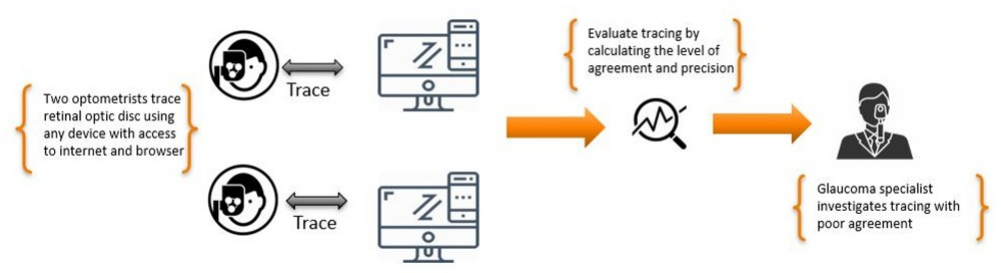
Tracing and data collection flow adopted in this study.

Two optometry doctors were involved in this study; their primary role was to trace the discs in the images obtained from the Drishti and ARIA datasets. These two datasets provided ground truth for discs obtained from the experienced ophthalmologists. Each optometrist involved in this study was assigned the sets of images (159 images in total) through the web portal, where they also traced the discs. The tracing was done independently and without consulting with each other. Fig 1 summarizes the overall process by which this stage of the research was conducted.

**Fig 2:**
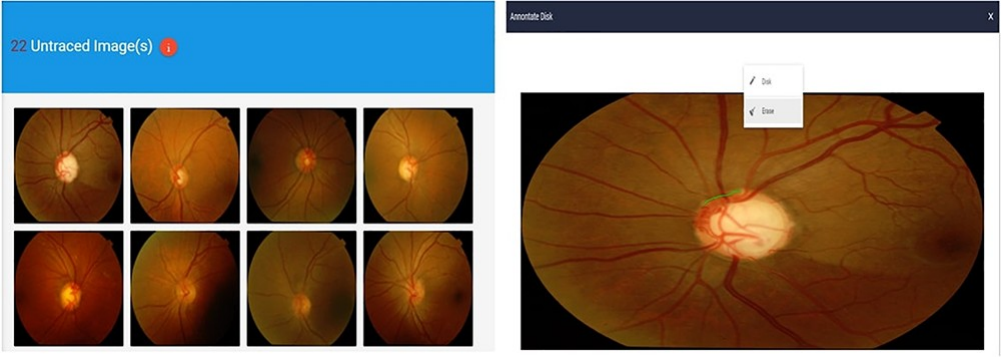
Web portal showing images assigned to the optometrists along with the pop up dialogue displayed once an image is pressed.

Once the pop-up model appears, the user can start tracing. An erase option is presented should the user wish to erase any of the tracings. We keep track of the time when the users start tracing and when this tracing is finished. Once the tracing is done, the user can click on the submit button, which will allow the storage of tracing information on a dedicated server. Users have the option either to trace the whole disc at once or in steps. Upon successful submission of the tracing, the traced image will be eliminated from the list of images on the tracing page and the number of untraced images at the top of the page, Fig 2, will also be decreased. Once the data had been stored, the traced images could be compared, and the ophthalmologist could investigate the reasons behind any significant disagreement mainly between optometrists.

### Statistical analysis

Three statistical evaluation techniques are adopted in this study, namely the Cohen’s kappa statistic [24] (to measure inter-agreement between the optometrists and between optometrists and available ground truth), the dice coefficient (to measure the precision of locating of the disc region) and average boundary distance (to measure precision in detecting disc border). The kappa calculation was calculated using Eq 1 where *Po* is the observed proportionate agreement and *Pe* is the probability of both optometrists saying either yes or no concerning whether a specific pixel should be considered as being in the disc region or not. When calculating the kappa value, we only included the area surrounding the disc. To do this we retrieved the lowest *x*, lowest *y*, highest *x*, and highest *y* values among the disc ground truth and tracing done by optometrists. Then we drew a bounding box which was used to calculate the kappa. There are various interpretations of kappa values; however, in this study, we adopted the most commonly used interpretation [25]. The cut off values are as follows:

1. below 0 ⇒ less than chance agreement
2. 0.01-0.2 ⇒ poor agreement
3. 0.21-0.40 ⇒ fair agreement
4. 0.41-0.60 ⇒ moderate agreement
5. 0.61-0.80 ⇒ substantial agreement
6. 0.81-0.99 ⇒ almost perfect agreement

$$\begin{array}{c} k=\frac{P_{o}-P_{e}}{1-P_{e}} \end{array}$$ (1)

**Fig 3:**
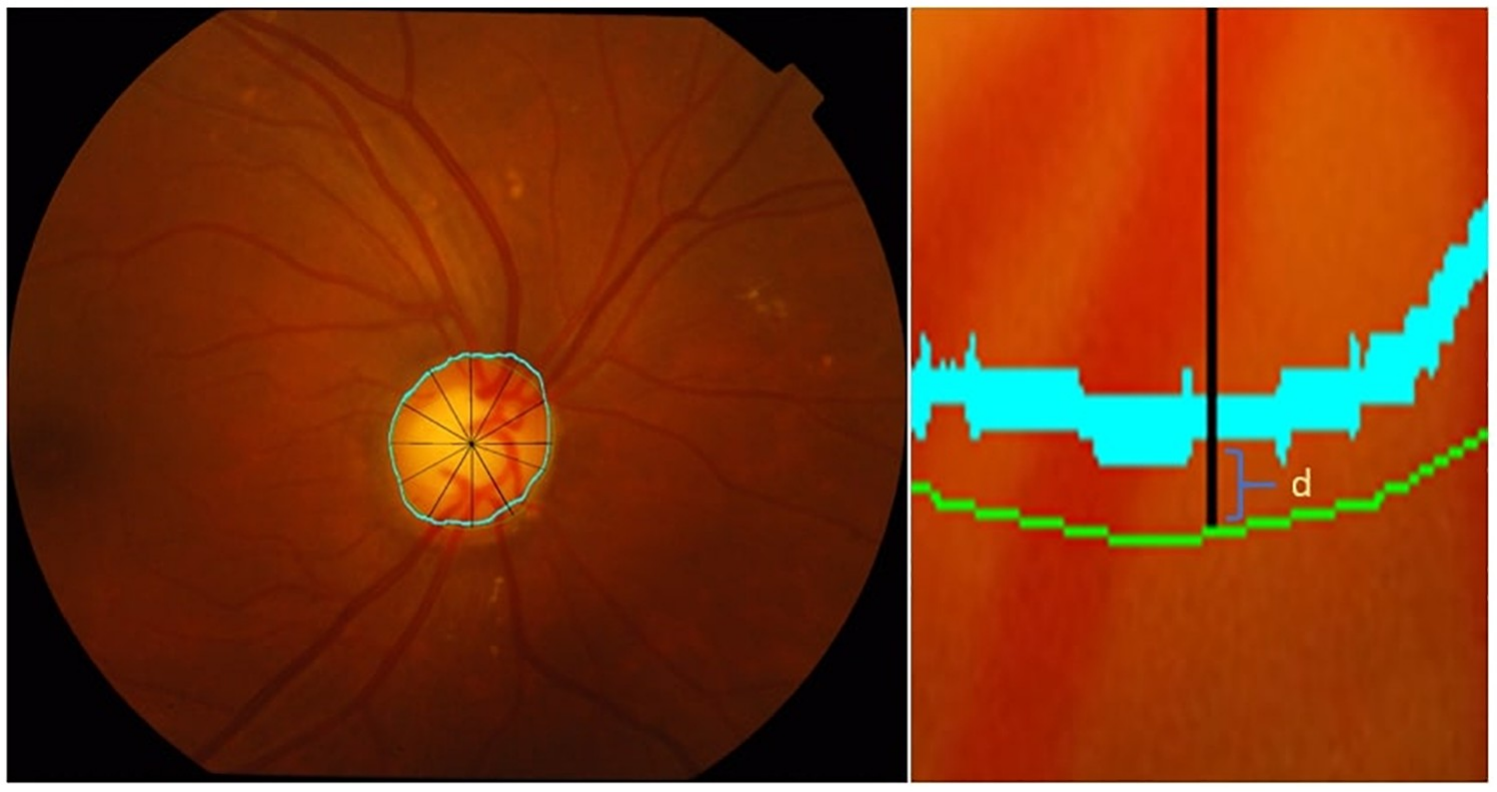
Retinal images showing lines draw from the centroid to expert (green) and optometrist (blue) traced boundary in different directions.

For the dice coefficient (*DC*) calculation, we used Eq 2, where *A* is the ground truth area and *B* is the area traced by the optometrist. A *DC* value of 1 means that the tracing of the optometrist agreed with the ground truth. However, *DC* does not indicate how precise the optometrists were in identifying disc boundary. Hence, we used another evaluation technique for this task known as the average boundary distance (*μ*_*d*_). This evaluation method compares the disc ground truth with the tracing done by each optometrist [26]. We used Eq 3, where $d_{k}^{g}$ and $d_{k}^{p}$ are the distance from the ground truth’s tracing centroid to the intersection point with the expert’s and optometrist’s tracings respectively at a given angle *k*. We calculated the distance in 12 directions namely 0°, 20°, 60°, 90°, 120°, 150°, 180°, 210°, 240°, 270°, 300°, and 330°. At each angle, a line is drawn from the centroid of the disc (*C*_*g*_). Then the intersection points between this line and ground truth, OD1, and OD2 tracing is detected at each of these 12 directions. An overview of the centroid and the 12 directions is shown in Fig 3. Moreover, we also show the distance difference between $d_{k}^{g}$ and $d_{k}^{p}$.

$$\begin{array}{c} DC=2*\frac{Area(A\cap B)}{Area(A)+Area(B)} \end{array}$$ (2)

$$\begin{array}{c} \mu_{d}=\frac{1}{n}\sum_{k=1}^{n}\left|d_{k}^{g}-d_{k}^{p}\right| \end{array}$$ (3)

While performing the analysis, we also noticed a calibration issue in that some of the tracings were misaligned. These misalignments were due to tracings deviating from their intended paths. While this issue could be fixed by erasing the misalignment, this process was fairly time-consuming. We hence used an approach called ellipse fitting to fix these misalignments by producing perfectly aligned ellipses; we then compared the tracings before and after the application of ellipse fitting. Another reason for using ellipse fitting is that various approaches claim that the discs tend to have an elliptic shape [6].

The least-square fitting approach identifies the smallest circle that can traverse all given traced pixels; specifically, we adopted the algorithm developed by Halir and Flusser [27]. Given Eq 4, where A,B,C,D,E, and F are the coefficients of the ellipse and *B*^2^ − 4*AC* < 0, generate an ellipse by minimizing the square of the algebraic distance of the points to the ellipsoid plane. This can be achieved by first calculating the design matrices as shown in matrices 5 and 6. These design matrices are then utilized to calculate the scattered plots as shown in Eq 7. The scatter plots are then reduced using the constrained coefficient in matrix 8 to produce the reduced matrix using Eq 9 where I and T represent the identity and transpose of the related matrices. The reduced scattered matrix is utilized to calculate eigenvectors and then extract the ellipse coefficients namely the center, width and height of the ellipse, and rotation angle. Additional information about the effectiveness of this approach with respect to other ones can be found in [27].

$$\begin{array}{c} Ax^{2}+Bxy+Cy^{2}+Dx+Ey+F=0 \end{array}$$ (4)

$$\begin{array}{c} D_{1}=(\begin{array}{ccc} x_{1}^{2} & x_{1}*y_{1} & y_{1}^{2}\\ \vdots & \vdots & \vdots \\ x_{n}^{2} & x_{n}*y_{n} & y_{n}^{2} \end{array}) \end{array}$$ (5)

$$\begin{array}{c} D_{2}=(\begin{array}{ccc} x_{1} & y_{1} & 1\\ \vdots & \vdots & \vdots \\ x_{n} & y_{n} & 1 \end{array}) \end{array}$$ (6)

$$\begin{array}{c} S_{1}=D_{1}^{T}*D_{1};S_{2}=D_{1}^{T}*D_{2};S_{3}=D_{2}^{T}*D_{2} \end{array}$$ (7)

$$\begin{array}{c} C_{1}=(\begin{array}{ccc} 0 & 0 & 2\\ 0 & -1 & 0\\ 2 & 0 & 0 \end{array}) \end{array}$$ (8)

$$\begin{array}{c} M=C1^{I}*\left(S_{1}-S_{2}*S_{3}^{I}*S2^{T}\right) \end{array}$$ (9)

Once the preceding steps were completed, we then measured the level of agreement between the two optometrists concerning the identification of the disc region both with and without ellipse fitting. We also investigated whether the level of agreement between the images taken from the Drishti and ARIA databases depended on the fundus status (i.e., normal, glaucoma, and diabetic retinopathy). Moreover, we investigated the level of precision with respect to the available ground truth for properly detecting the disc regions between the two datasets and among different retinal image statuses. Further, we study the level of agreement with regard to correctly detecting the disc boundary and how precise the optometrists were in doing so. All data generated in this study is publicly available for use by researchers [23].

## Results

**Fig 4:**
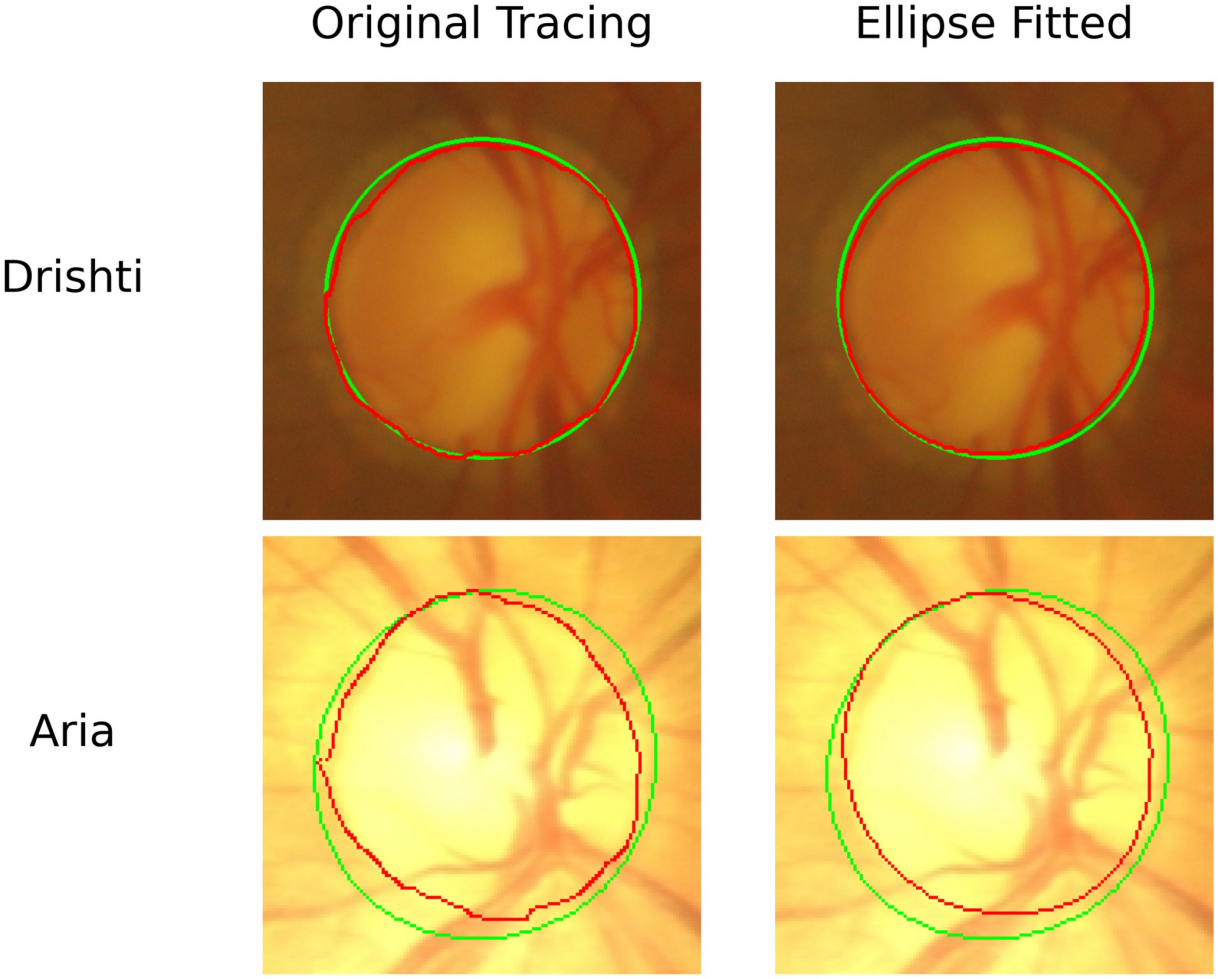
Images obtained from the Drishti and ARIA datasets showing improved disc tracing when ellipse fitting is applied. The first row represents an image from the Drishti dataset before and after applying ellipse fitting. The second row is similar to the first one but for an image obtained from the Aria dataset. The green tracing represents the ground truth and the red represents the tracing performed by the optometrist. The application of Ellipse fitting led to an increase of DC from 96.80% to 98.10% for the Drishti image from 95.60% to 96.10% for the Aria image.

**Fig 5:**
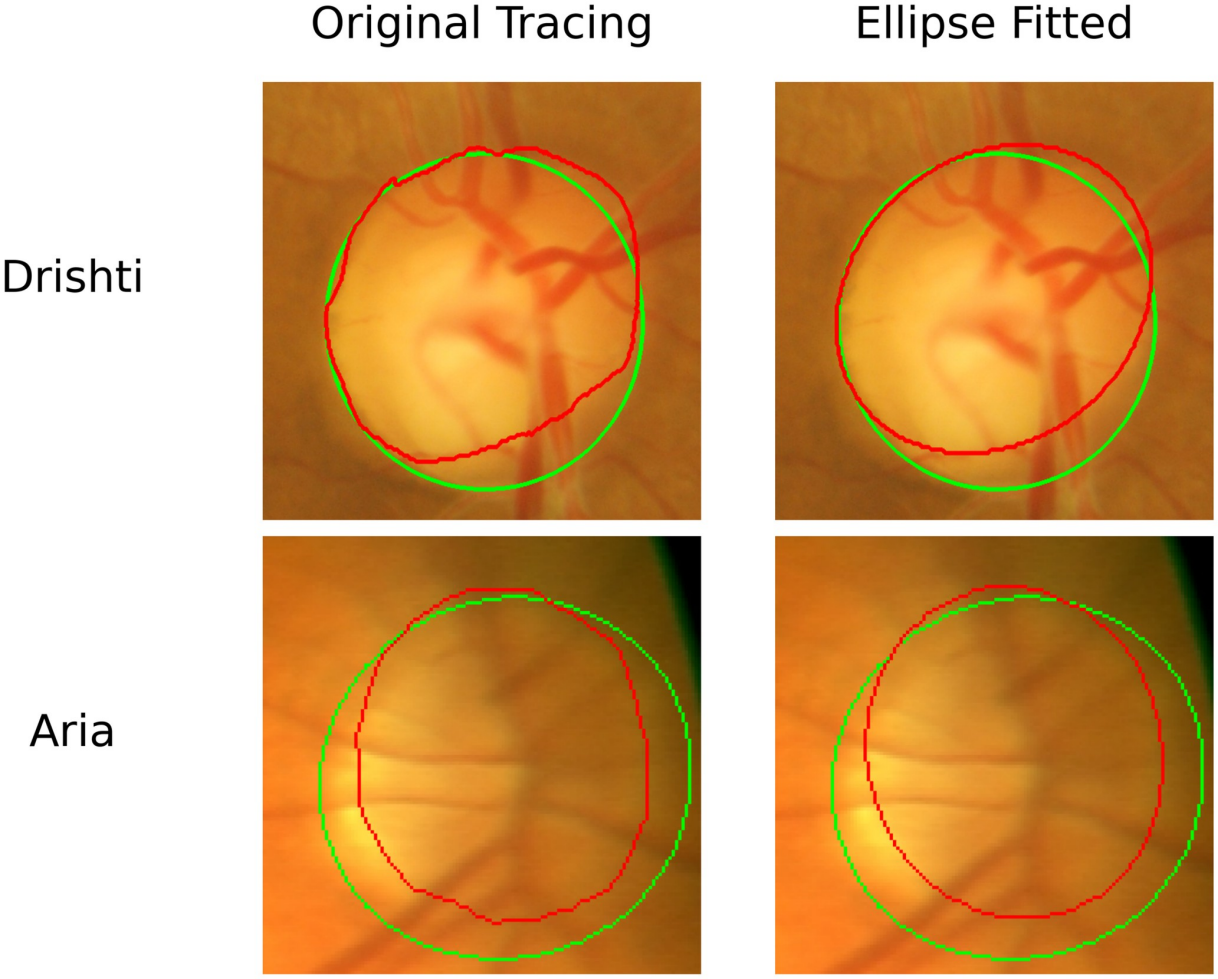
Images obtained from the Drishti and ARIA datasets showing ellipse fitting lead to lower precision in the traced disc region. The first row represents an image from the Drishti dataset before and after applying ellipse fitting. The second row is similar to the first one but for an image obtained from the Aria dataset. The green tracing represents the ground truth and the red represents the tracing performed by the optometrist. The application of Ellipse fitting led to a decrease of DC from 90.59% to 89.35% for the Drishti image from 86.28% to 84.73% for the Aria image.

In this section, we discuss various analysis techniques applied on the tracing performed by OD1 and OD2 along with comparing them with ground truth. A summary of the results before and after applying ellipse fitting can be seen in Tables 1 and 2 respectively. Additionally, Figs 4 and 5 demonstrate the performance of ellipse fitting in fixing the misalignment in the tracing of the optometrists. We realized that applying ellipse fitting does not always improve the precision in tracing. For instance, in Fig 4 when applying ellipse fitting the DC has increased 96.8% to 98.1% for the Drishti image while for the Aria image, the DC has increased from 95.6% to 96.1%. However, this was not the case in Fig 4 where the DC for the Drishti after applying ellipse fitting decreased from 90.59% to 89.35% and from 86.28% to 84.73% for the Aria image. We should emphasize that the Drishti dataset is mainly for glaucoma while the Aria dataset is for patients with diabetic retinopathy.

**Table 1:** The values for DC, kappa, and boundary when comparing OD1 with OD2, OD1 with ground truth (G), and OD2 with ground truth (G) before applying ellipse fitting.

	Dice Coefficient		Kappa (region)		Kappa (border)		Boundary (pixels)	
	Drishti	Aria	Drishti	Aria	Drishti	Aria	Drishti	Aria
OD 1 vs OD 2	-	-	0.86	0.84	0.09	0.15	6.60	1.80
OD 1 vs G	0.95	0.92	0.86	0.80	0.015	0.09	6.90	3.20
OD 2 vs G	0.95	0.93	0.85	0.80	0.02	0.05	7.70	3.40

**Table 2:** This table summarizes the values for DC, kappa, and boundary when comparing OD1 with OD2, OD1 with ground truth (G), and OD2 with ground truth (G) after applying ellipse fitting.

	Dice Coefficient		Kappa (region)		Kappa (border)		Boundary (pixels)	
	Drishti	Aria	Drishti	Aria	Drishti	Aria	Drishti	Aria
OD 1 Vs OD 2	-	-	0.88	0.86	0.18	0.28	5.70	1.30
OD 1 vs G	0.95	0.92	0.85	0.79	0.13	0.102	6.90	3.20
OD 2 vs G	0.95	0.93	0.84	0.79	0.02	0.06	7.70	3.40

### Tracing agreement

**Fig 6:**
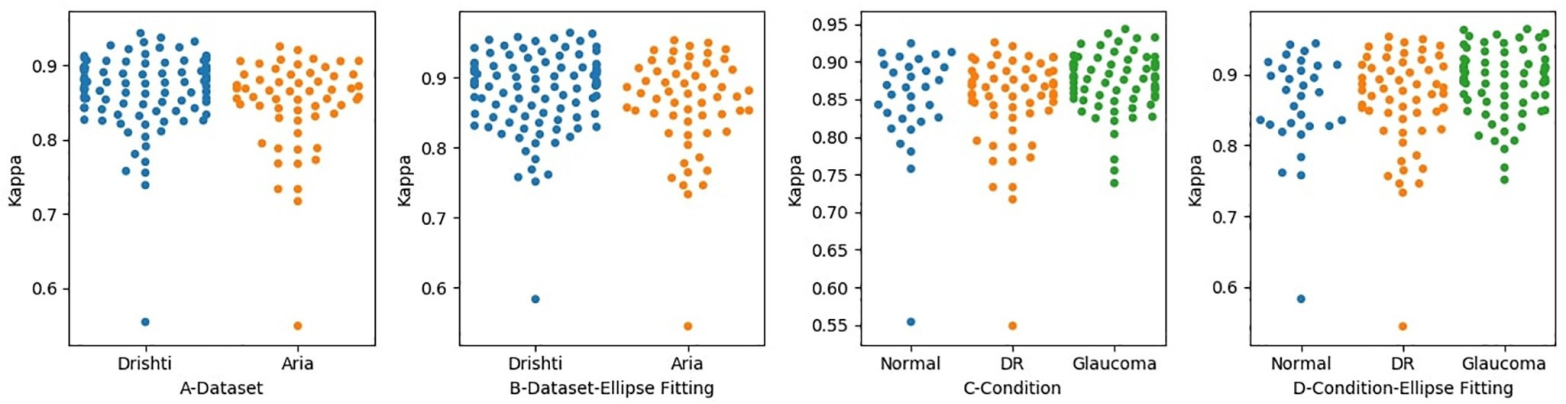
The level of agreement between the optometrists when identifying disc region. Figures A and B Show the Level of Agreement in each Dataset. Figures C and D show the Level of Agreement in Regard to Retinal Condition.

To measure the level of agreement between the two optometrists OD1 and OD2, we calculated the kappa value for the level of agreement between the optometrists in disc tracing. We calculated the level of agreement in terms of locating the disc region in each dataset. Before the application of ellipse fitting, the level of agreement for the Drishti dataset was 0.86, while that for the ARIA dataset 0.84, which were both almost perfect. After applying ellipse fitting, the level of the agreement changed slightly, with the level for Drishti being 0.88 and for ARIA 0.86, as shown in Fig 6. As can be seen in this figure, after applying ellipse fitting, we realized there was an improvement in some images in the Drishti dataset, as their kappa values increased from 0.94 to 0.96; meanwhile, the kappa values for some images from the ARIA set increased from 0.92 to 0.95. We also realized the kappa value for the image with the lowest agreement score in the Drishti dataset improved from 0.55 to 0.59. While this was not a significant improvement, it shows the effect of misalignment in tracing and how ellipse fitting can be used to address this issue in some images but not to all.

We took the analysis one step further by investigating whether there was a difference in determining the disc region as a result of retinal status. We measured the level of agreement with respect to the presence of retinal conditions, namely glaucoma, diabetic retinopathy (DR), and normal eyes (see Fig 6A and 6B). We compared the results with and without ellipse fitting. The levels of agreement before applying ellipse fitting for glaucoma, DR, and normal were 0.87, 0.84, and 0.84, respectively, which are slightly lower than the levels achieved after applying ellipse fitting, which were 0.91, 0.86, and 0.85, respectively. The major improvement has occurred in the images related to glaucoma. It is also clear how the distribution of kappa values for the images changed before and after ellipse fitting, as can be seen in Fig 6C and 6D. This change is reflected by the fact that the image with the lowest level of agreement in the Drishti sample has a better level of agreement after the application of ellipse fitting. Such improvement in the level of agreement is mainly because one of the ODs encountered calibration issues while tracing the disc. However, the application of ellipse fitting did not always lead to an improvement in results, check Fig 5, and highly dependant on how much miss alignment was produced by the optometrist when tracing the disc.

**Fig 7:**
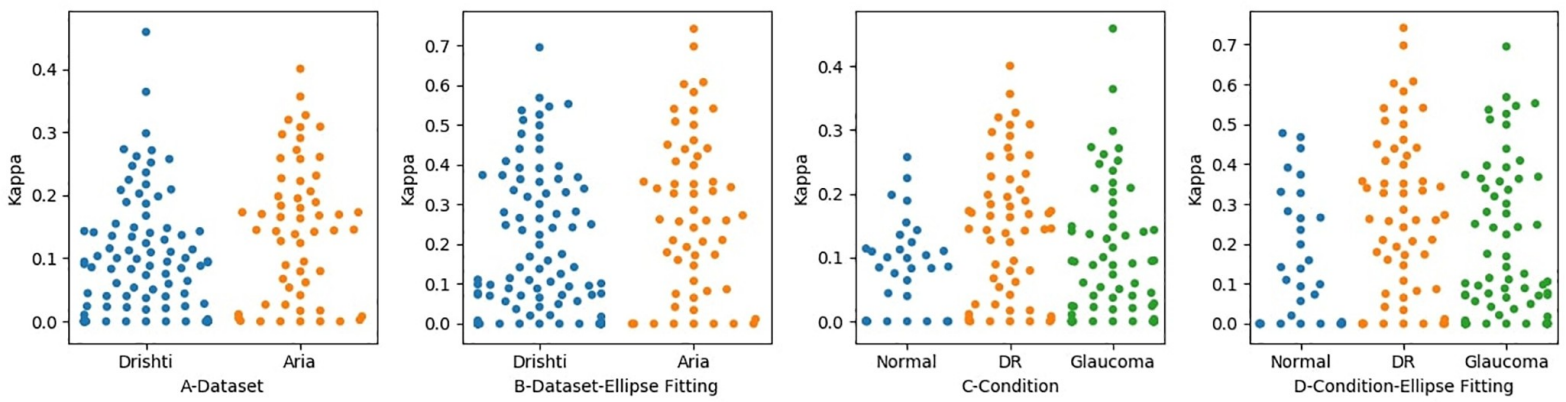
The level of agreement between the optometrists when identifying disc region. Figures A and B Show the Level of Agreement in each Dataset. Figures C and D show the Level of Agreement in Regard to Retinal Condition.

Measuring the level of agreement with regard to detecting the disc regions was found not reliable in determining the level of agreement in detecting disc borders. We also measured the level of agreement between OD1 and OD2 in locating the disc border. We first checked the level of agreement based on the dataset and then based on retinal condition, as shown in Fig 7A and 7B. The levels of agreement between the two optometrists before applying ellipse fitting to the Drishti and ARIA datasets were 0.09 and 0.15 respectively, which are lower than the levels of agreement after applying ellipse fitting, which were 0.18 and 0.28, respectively. Thus, the level of agreement after applying ellipse fitting improved from poor to fair for the ARIA dataset but remained poor for the Drishti. We also found that the level of agreement with regard to the ARIA dataset was slightly better than that for the Drishti dataset before applying ellipse fitting; this is mainly because of the presence of PPA in glaucomatous images. The levels of agreement for some images in the Drishti and ARIA datasets improved from moderate to substantial agreement, which is considered a significant improvement. However, in some images, the level of agreement decreased to zero; this likely occurred due to misalignment towards the disc center and/or because ellipse fitting takes the minimal point, meaning that the disc boundary will change.

When we measured the level of agreement in detecting the disc region, we also measured the level of agreement in detecting the disc border based on retinal status (see Fig 7C and 7D). The levels of agreement for glaucoma, DR, and normal retinal images before applying ellipse fitting were 0.09, 0.15, and 0.08, respectively, which are considered to be very poor values and are lower than the levels of agreement achieved after the application of ellipse fitting (0.19, 0.3, and 0.2 for glaucoma, DR, and normal images, respectively, with these values being considered to represent fair levels of agreement). Applying ellipse fitting helped to improve the level of agreement from poor to fair in boundary identification, particularly for images related to glaucoma. However, this level of agreement is still low and may have a negative impact on patient referrals, especially for those with glaucoma. We can also realize that glaucomatous images have the lowest level of agreement among other images.

### Tracing precision

Measuring inter-agreement between the optometrists made it possible to obtain a value for the level of agreement, but it did not indicate how precise the optometrists were. To measure the level of precision in detecting the disc region, we calculated the dice coefficient using Eq 2. We calculated the dice coefficient for OD1 and OD2 with respect to the available ground truth. The dice coefficient is useful when measuring precision as dependent on the volume of a traced object but not when measuring precision in detecting an object’s boundary. For this task, we used another approach called average distance boundary as discussed in section 1. We applied Eq 3 to each image for the tracing done by each optometrist.

**Fig 8:**
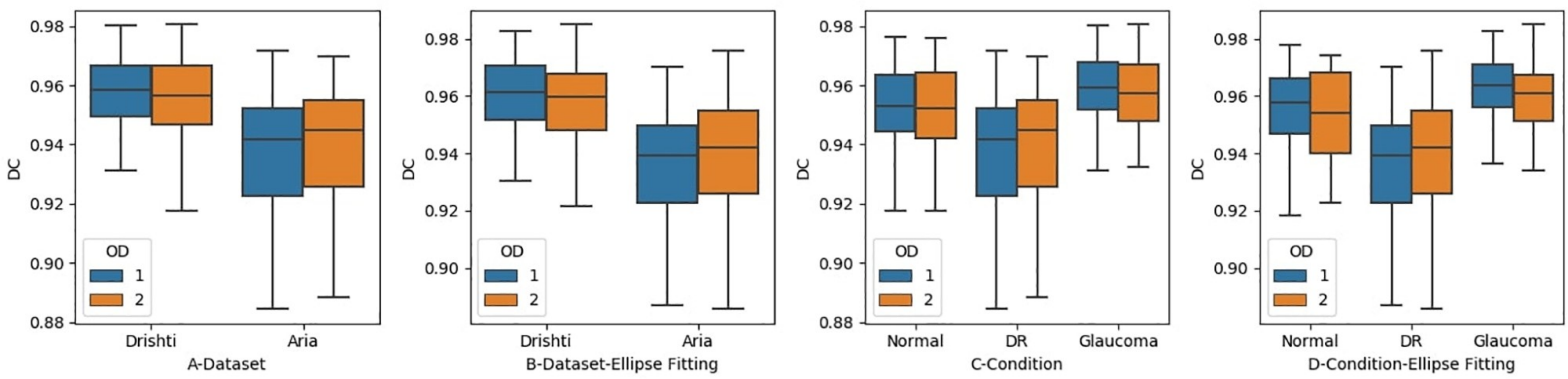
The level of precision between the optometrists when identifying disc region. Figures A and B Show the Level of Precision in each Dataset. Figures C and D show the Level of Precision in Regards to Retinal Condition.

We first measured the level of precision in detecting the disc region in each dataset, regardless of the retina status, before and after applying ellipse fitting, as shown in Fig 8A and 8B. The DC values obtained by OD1 were 0.95 for the Drishti dataset and 0.92 for the ARIA dataset, while OD2 achieved 0.95 for the Drishti dataset and 0.93 for the ARIA dataset. The level of precision did not improve significantly after the application of ellipse fitting, as it stayed almost the same (see Fig 8). However, images with low levels of tracing precision improved after the application of ellipse fitting. Both optometrists performed almost the same in identifying disc region.

**Fig 9:**
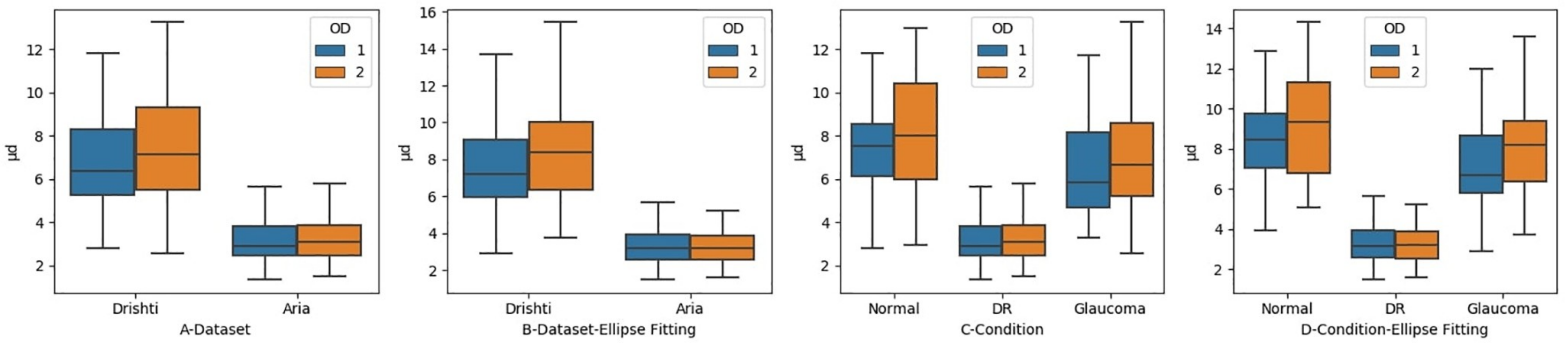
The level of precision between the optometrists when identifying disc border. Figures A and B Shows the Level of Precision in each Dataset. Figures C and D show the Level of Precision in Regards to Retinal Condition.

We also investigated whether the level of precision in terms of detecting the disc region was affected by retinal status, both before and after the application of ellipse fitting, as shown in Fig 8C and 8D. The levels of precision for OD1 before applying ellipse fitting were 0.95, 0.92, and 0.94 for glaucoma, DR, and normal retinal images, respectively, while those for OD2 were 0.95, 0.92, and 0.95. However, ellipse fitting did not lead to improvement in the precision, as the values remained roughly the same. We can also realize that ellipse fitting helped to slightly improve the tracing precision for OD2. However, for some images, ellipse fitting has a negative effect when being compared with the ground truth. This is depending on where the misalignment occurred, and how frequently it happened. Overall, we can see improvement in the lower and upper bounds in Fig 9 before and after applying ellipse fitting. Moreover, the levels of precision in glaucoma and normal images for both optometrists remained almost the same after the application of ellipse fitting, but we can realize improvements in the upper and lower bounds along with the median value.

In addition to measuring the level of agreement in detecting the disc border, we also measured the level of precision in correctly identifying the disc boundary by OD1 and OD2 both before and after applying ellipse fitting to each dataset, as shown in Fig 9A and 9B. Instead of using the dice coefficient, we used the average boundary distance measure described earlier to measure boundary tracing precision with respect to the available ground truth. The average boundary distance before applying ellipse fitting for OD1 were 6.9 and 3.2 pixels for the Drishti and ARIA datasets, respectively, while those for OD2 were 7.7 and 3.4 pixels. These results revealed some information that could not be discovered using only DC values. DC values showed perfect precision in identifying disc region, but this was not the case when trying to check for the boundary. Moreover, in some cases, the difference between ground truth and optometrist tracing distance reached 53 pixels. It can thus be concluded that the optometrists were unable to correctly detect the disc boundary mainly in the Drishti dataset. This can also be correlated to a poor level of agreement achieved in detecting disc boundary. The application of ellipse fitting did not affect the overall results for μd. Moreover, the results showed poor tracing precision in the glaucomatous dataset (Drishti). OD1 performed better than OD2 and this can be clearly seen in Fig 9. Such observations can not be achieved without using evaluations similar to the boundary distance one.

A similar measurement was applied to gauge the average boundary distance in terms of determining retinal image status for the two optometrists before and after applying ellipse fitting (Fig 9C and 9D). The levels of boundary distance for OD1 before applying ellipse fitting were 6.6, 3.2, and 7.4 for glaucoma, DR, and normal retinal images, respectively, while those for OD2 were 7.3, 3.4, and 8.6. We did not observe significant improvement in the overall results after applying ellipse fitting. Actually, ellipse fitting negatively affected the tracing done for some images especially for those traced by OD2. The overall performance in terms of correctly detecting the disc border in all retinal conditions was low; indeed, in some cases, it was as low as zero. Images with DR had the highest level of precision while those with glaucoma had the lowest as can be observed from high average boundary distance. This is mainly related to the level of subjectivity when trying to distinguish the disc border from PPA.

## Discussion

This study evaluated the level of agreement and precision for tracing the disc in fundus images. This involved the development of a customized web portal for disc annotation. We found that optometrist tracings showed high levels of agreement for identifying the disc region. However, there was poor agreement for correctly identifying the disc boundary. The application of ellipse fitting helped in adjusting for misalignment in some images.

Two optometrists OD1 and OD2 produced a set of disc and disc-boundary traces of fundus images. A glaucoma specialist went over the tracings and found that the two optometrists had a great variance in detecting the disc boundary and distinguishing it from PPA in the inferior and superior regions of the disc in various images. One of the reasons for this variance is that the disc border was not very clear in certain images, and hence the bend of blood vessels was used instead, which does not allow the actual disc border to be identified correctly in all cases. Another reason was the calibration challenge faced by optometrists when tracing the whole disc at once. Although an eraser was provided with which to remove incorrect tracing, this feature did not prevent this issue from occurring. We used ellipse fitting to address misalignments in tracing, which helped to improve the levels of agreement and precision for some images. The low resolution and poor disc visibility in some of the images were additional reasons for the poor performance of the optometrists. In some of the images obtained from the ARIA dataset, the disc border was not very clear, and it was occasionally blurred by background illumination. However, optometrists performed better in locating disc boundary in images from the ARIA dataset as compared with the Drishti dataset.

On average, OD1 took 80 seconds to completely trace the disc, which was longer than OD2’s 30 seconds. In general, OD1 performed better than OD2 in tracing the disc, which is reflected in a higher DC values and lower average boundary distance achieved than that of OD2, particularly with regard to the disc border. Both optometrists achieved high kappa and DC in identifying disc regions, but this was not the case when locating the disc boundary especially in the images from the glaucomatous dataset.

In general, the portal is a great tool for disc tracing instead of using a desktop application. Users can use any device with an internet browser that has access to the internet. Once access is established they can start tracing the images assigned to them and share their results. We also showed that applying ellipse fitting to tracings is not always helpful. The effectiveness of ellipse fitting is highly dependent on the misalignment of tracing caused by the user and in some cases the shape of the disc is not close to elliptic. One of the limitations of this study is that, for some of the images, the optometrists found it difficult to use the pencil and eraser, which led to more time being spent on specific images. Future work could be done using a customized object with a rotation feature, which can be used to compare participants’ results. Another limitation is that the system does not currently provide live feedback, which is a feature that we intend to add. We would like to expand this system to include annotation of other retinal anatomical objects, such as the fovea and PPA. Additionally, we would like to develop a semi-supervised approached that could help in speeding up the tracing process. While the system has the potential to be applied in any field that involves tracing, our research mainly focuses on the field of ophthalmology, in particular glaucoma.

In this study, the levels of precision and agreement were very low when it comes to correctly detect the disc border especially when working with glaucomatous retinal images. Hence, developing an automated approach that could assist in correctly detecting the disc border in a precise, consistent, and rapid fashion is very important in this field. Such an approach would have the potential to be helpful in detecting glaucomatous discs, influencing decisions concerning treatment, offering appropriate referrals, as well as in other areas. The developed portal in this study could serve as a model for generating sufficient ground truth to develop an automated system for identifying cup to disc ratio and helping to improve the identification of individuals who may have glaucoma, allowing for more timely referrals and management. We also demonstrated that there is a degree of subjectivity in tracing the disc. Using the average boundary distance evaluation approaches revealed information related to optometrists’ precision in disc boundary tracing that was not revealed when using DC. Using kappa and average boundary distance identified where disagreement is happening which we then investigated.
